# Emerging trends in bone marrow organoid research: From hematopoietic microenvironment reconstruction to translational and regenerative theranostic applications

**DOI:** 10.7150/thno.137874

**Published:** 2026-07-13

**Authors:** Heebin Park, Jaemin Jeong

**Affiliations:** Department of Biohealth Convergence, College of Science and Convergence Technology, Seoul Women's University, Seoul, 01797, Republic of Korea.

**Keywords:** biomaterials, bone marrow, bone marrow environment, disease model, organoids

## Abstract

Bone marrow organ is characterized as a dynamic tissue with a complex microenvironment wherein hematopoietic stem cell (HSC) homeostasis is maintained, and the generation of various hematopoietic cell subsets is regulated. Organoids technology has been applied as an alternative tool for modeling complex tissue microenvironments in a laboratory setting through the self-organization of cells. Recent studies have established a platform capable of studying complex cellular interactions by generating bone marrow organoids (BMOs) from iPSCs. Moreover, BMOs can emulate the architecture observed of the bone marrow, including the various cell types, containing vascular-like networks, HSCs, mesenchymal stromal cells (MSCs), and mature hematopoietic cells. This review aimed to summarize how BMOs can provide foundational data essential for understanding similarities in the microenvironment and the pathological mechanisms underlying bone marrow diseases, as well as for developing new treatments. Furthermore, BMO systems represent a cutting-edge platform for studying hematopoiesis, disease mechanisms, and therapeutic screening, highlighting the recent trend toward physiologically relevant organoid-based models in regenerative and hematopoietic research.

## Introduction

The bone marrow is a hematopoietic organ that contributes to blood cell production, immune regulation, and niche-mediated support for HSCs 1-4 and supports continuous hematopoiesis throughout life 1, 2. An important concept in bone marrow physiology is the HSC niche, a specialized microenvironment that regulates the self-renewal, quiescence, and differentiation of HSCs 1, 5. This niche comprises of diverse cellular and molecular components that interact through complex signaling networks and regulate the fate and function of hematopoietic stem and progenitor cells (HSPCs) 6, 7. The interactions between hematopoietic cells and multiple types of bone marrow niche cells, such as MSCs, endothelial cells (ECs), and osteoblastic cells, ensure bone marrow homeostasis 5, 8. Moreover, the interactions between cells within the bone marrow microenvironment highlight that hematopoiesis occurs within a complex, precisely regulated environment.

**Figure 1:**
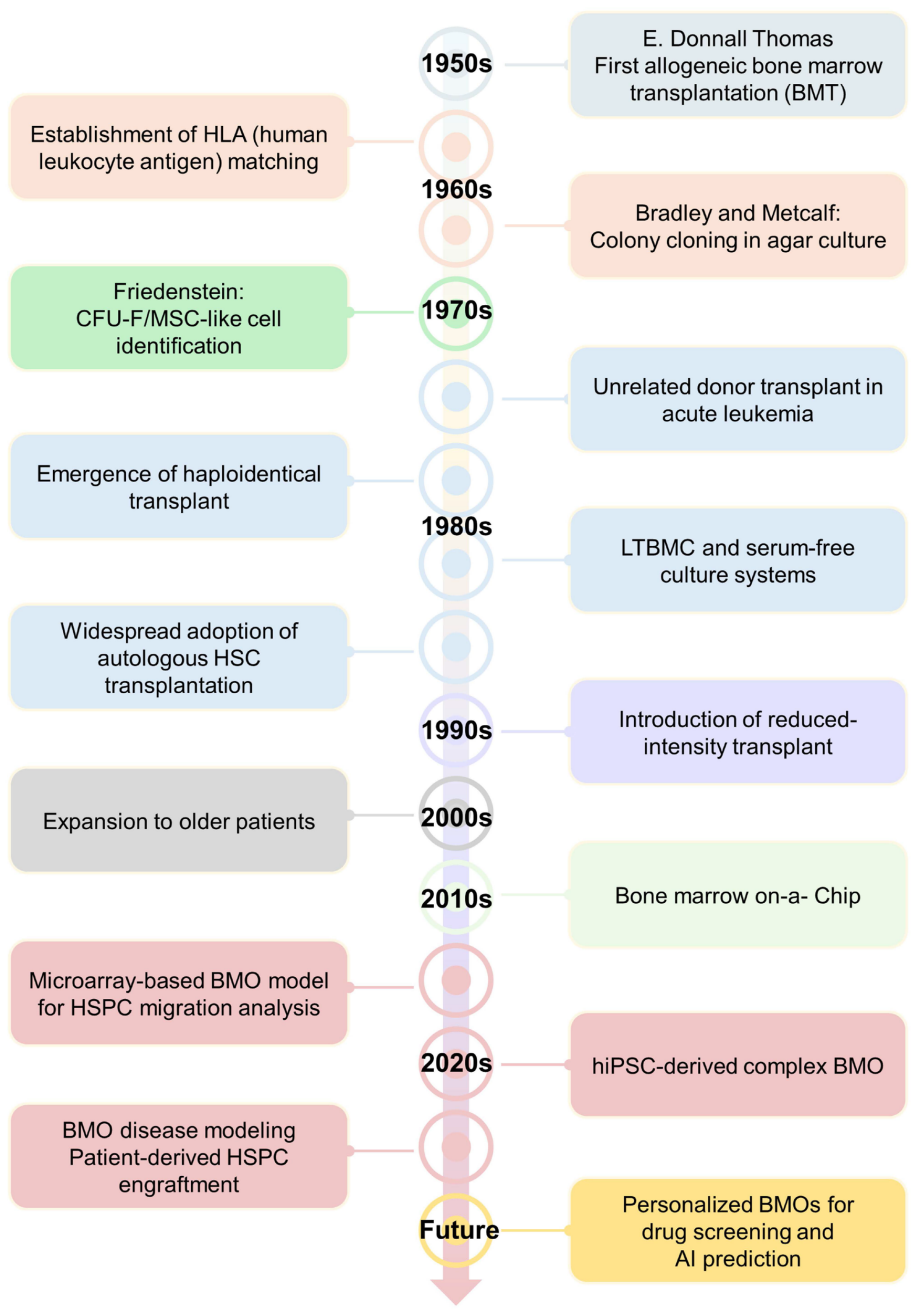
** History and major milestones in bone marrow research.** A timeline showing the major milestones in bone marrow research, from early transplantation studies and *in vitro* bone marrow culture systems to recent advances in BMO models. Early research has focused on improving HSC memory and establishing experimental platforms for *in vitro* bone marrow research, while recent research has expanded toward the microphysiological and organoid based modeling.

Bone marrow research has evolved as an important field for understanding HSCs, hematologic physiology, and related diseases. Although various technologies have been established to reduce immune rejection and improve transplantation outcomes, the development of *in vitro* models that can reproduce the complex bone marrow microenvironment in the laboratory is essential. Early colony-forming studies using mouse bone marrow cells cultured in semi-solid agar, the identification of colony-forming unit-fibroblasts (CFU-F), and the development of long-term bone marrow culture and serum-free culture systems provided the conceptual and technical foundation for *in vitro* bone marrow research (Figure 1) 9-11. In parallel, advances in hematopoietic cell transplantation, including allogeneic, unrelated donor, autologous, and haploidentical transplantation, emphasized the importance of the bone marrow microenvironment in determining stem cell engraftment and hematopoietic recovery (Figure 1) 12, 13. Subsequently, microphysiological systems such as bone marrow-on-a-chip partially reconstructed the vascular and stromal microenvironmental functions *ex vivo*, further extending this line of research and ultimately laying the foundation for future BMO studies (Figure 1) 14.

Since 2020, BMO research has evolved from proof-of-concept platforms to more sophisticated human-derived models (Figure 1). Recent studies have shown that BMOs can recapitulate the essential features of bone marrow biology, including vascular-like networks, stromal organization, hematopoietic progenitor populations, and multilineage differentiation (Figure 1) 4, 15-18. In particular, the development of microarray-based BMOs for analyzing HSPC migration represented an important milestone, followed by the establishment of human BMO systems capable of disease modeling and therapeutic target validation 17. Furthermore, hiPSC-derived BMOs can be effectively used as a platform for reconstructing vascular and stromal microenvironments, while patient-derived HSPC engraftment models demonstrate the potential for the clinical and translational expansion of this field 4, 15, 16. These advances have positioned BMOs not only as helpful tools for studying microenvironment-dependent hematopoiesis and bone marrow pathophysiology but also as promising platforms for patient-specific disease modeling, therapeutic screening, and regenerative medicine applications.

The present study focused on the development of BMOs since 2020. We discussed major advances in BMO generation technologies, their applications in hematopoietic function and disease modeling, and the remaining challenges and future directions for establishing BMOs systems with both physiological relevance and clinical usefulness. Building upon these advancements, this review article aimed to summarize the current progress in BMOs research, highlight its applications in modeling hematopoietic function and disease pathology, and discuss new challenges and future directions toward translational and regenerative applications.

## Cellular components of the bone marrow niche relevant to organoids design

### Hematopoietic stem cells (HSCs)

The bone marrow stem cell niche provides a specialized microenvironment wherein the HSCs reside. Within this niche, HSC fate decisions are regulated by both intrinsic and extrinsic mechanisms 2, 19, 20. HSCs are functionally defined as cells that can sustain over the long term and reconstitute the hematopoietic system following an injury or ablation 21. This definition is based on the ability of HSCs to undergo self-renewal and facilitate the production of all hematopoietic lineages, including both myeloid and lymphoid blood cells, indicating their multipotent differentiation capacity 22, 23. Within the bone marrow, HSCs tend to exhibit long-term hematopoietic reconstitution, which refers to the stable maintenance of the hematopoietic system throughout the lifespan rather than transient hematopoietic activities. This long-term maintenance is enabled by specific anatomical regions within the bone marrow, which are collectively referred to as the HSC niche. Recent studies have reported that many HSCs are located near sinusoidal vessels, suggesting that the perivascular region, along with the endosteal niche, can serve as an important microenvironment for the localization and maintenance of HSCs 24, 25. Within this niche, ECs and stromal cells cooperate to create a microenvironment essential for hematopoietic homeostasis 2, 6, 25, 26. Indeed, bone marrow contains diverse microenvironments, and HSCs primarily reside in the perivascular region, where ECs and stromal cells interact closely to create an environment that supports hematopoietic regulation 27, 28. Within this niche, the CXCL12–CXCR4 signaling axis plays a central role in maintaining HSC quiescence and guiding their spatial localization 29, 30. CXCL12-abundant reticular (CAR) cells are a subset of bone marrow stromal cells that secrete high levels of CXCL12, are distributed in a reticular pattern around the HSCs niche and regulate the establishment and maintenance of HSCs 15, 31-33. Simultaneously, SCF regulates HSCs adhesion and motility, acting in concert with CXCL12-mediated chemotactic cues to maintain proper stem cell positioning and retention in the bone marrow 29, 34, 35. Notably, membrane-bound SCF expressed by stromal and endothelial cells stabilizes the niche, ensuring long-term maintenance of the quiescent HSCs pool 35-37. Recent advances in three-dimensional (3D) BMO models have allowed the faithful reconstruction of this regulatory network 4, 15, 16, 18. Importantly, organoid-derived HSPCs have been shown to differentiate into myeloid and lymphoid progenitors, including granulocytes, monocytes, macrophages, and megakaryocytes 4, 15, 16, 18. In line with this, HSCs facilitate the production of myeloid lineage cells in the bone marrow, including erythrocytes, neutrophils, eosinophils, basophils, monocytes, and platelets, while differentiating into lymphoid lineage cells such as T cells, B cells, NK cells, and plasma cells (Table 1). Within these systems, HSCs reside in the perivascular regions and exhibit gene expression patterns and migratory behaviors that are consistent with *in vivo* marrow physiology. Furthermore, the re-establishment of CXCL12–CXCR4 and SCF-dependent interactions within the organoid validates the formation of a functional hematopoietic niche capable of supporting physiological HSC maintenance and multilineage differentiation 4.

### Mesenchymal stromal cells (MSCs)

MSCs are a central component of the bone marrow microenvironment and serve as key regulators of hematopoietic homeostasis 5, 8. These multipotent cells produce extracellular matrix (ECM) proteins such as collagen, laminin, and fibronectin, which constitute the structural framework necessary for the compartmentalization and organization of hematopoietic cells and ECs in the bone marrow microenvironment 4, 38, 39. Such ECM-based architectures allow for the spatial organization of the bone marrow niche and provide the foundation for sustaining stable hematopoietic function 40. MSCs also regulate stem cell function and the organization of the cells within the niche through direct interactions and paracrine signaling with hematopoietic cells and ECs 41. Moreover, MSCs that are localized in the perivascular niche secrete CXCL12, which promotes HSC retention within the niche via CXCR4 signaling and helps maintain their quiescence 42, 43. They also produce ANG-1, which is received through the Tie2 receptor on HSCs to reinforce quiescence and simultaneously promote vascular stabilization within the niche 42, 44. MSCs tends to cooperate with ECs to promote angiogenesis, support HSC regeneration in a transplantation setting, or regulate the stability of the vascular niche 45, 46. In organoid models, they self-organize into stromal and vascular compartments, recapitulating their *in vivo* role in regulating hematopoietic activities 4, 15. Within the bone marrow niche, MSCs integrate mechanical and biochemical cues to maintain hematopoietic homeostasis and orchestrate the formation of a physiologically relevant BMO microenvironment.

### Endothelial cells (ECs)

ECs constitute the vascular compartment of the bone marrow niche, which serves as a structural scaffold and paracrine regulator that maintains hematopoietic homeostasis 19. They secrete angiocrine signals such as VEGF, and FLT4, which regulate HSC self-renewal, differentiation, and mobilization 47, 48. The vascular niche contains sinusoidal and arterial endothelial compartments 48. Sinusoidal ECs tend to secrete vasculogenic factors, such as CXCL12, SCF, and VEGFA, and facilitate the maintenance and regeneration of HSCs through interactions mediated by adhesion molecules, including E-selectin 20, 49, 50. In the bone marrow, sinusoidal ECs are characterized by high permeability and a relatively elevated ROS environment, while they secrete factors such as CXCL12, VCAM1, and E-selectin to promote the activation, regeneration, and differentiation of HSCs 51, 52. In contrast, arteriolar ECs are associated with a relatively low-ROS microenvironment and support the long-term dormancy and maintenance of HSCs by providing antiproliferative and Notch signals, including DLL4 and JAG-1 53, 54. These vascular EC subtypes establish distinct microenvironmental niches that regulate the localization and functional status of HSCs. Recently, 3D BMO systems have allowed for the partial *in vitro* reconstruction of these vascular niches. In hiPSC-derived BMOs and MSC precursor-EC co-culture-based BMO models, ECs self-organize into arterial-like networks and reproduce major vascular and stromal interactions which constitute CXCL12⁺ perivascular cells, LepR^+^ and Nestin^+^ stromal populations, and megakaryocytes positioned near sinusoid-like vessels 4, 15, 18, 31, 32. These niches recapitulate essential endothelial and stromal signaling, supporting the long-term maintenance of HSPCs and multilineage hematopoiesis without exogenous cytokines 4. The ECs within 3D bone marrow niches serve as niche regulators, integrating structural, metabolic, and biochemical signals that are crucial for hematopoietic regulation.

## The evolution of bone marrow organoid systems

### Early stromal–vascular co-culture models for HSPC homing

**Figure 2:**
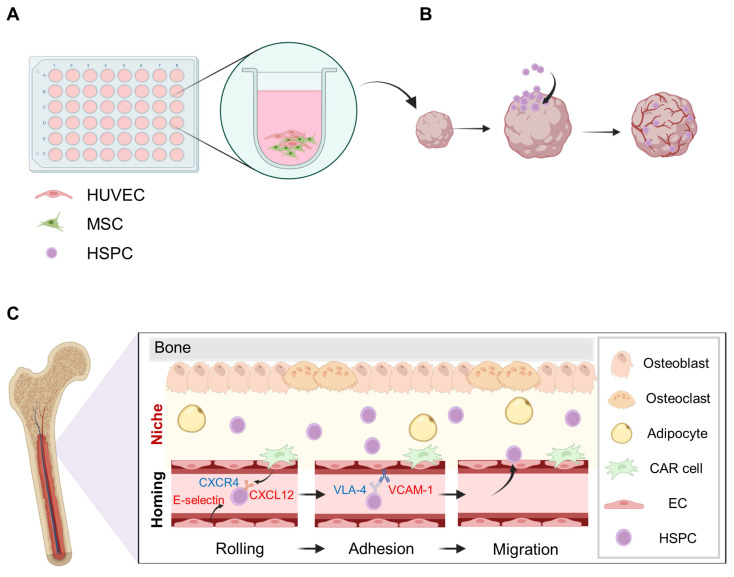
** A 3D stromal–vascular co-culture system for BMO generation and HSPC engraftment. (A)** The BMOs were generated by co-culturing MSCs and human umbilical vein endothelial cells (HUVECs). **(B)** Following transplantation, the HSPCs were delivered through the bloodstream and successfully engrafted into the organoids. **(C)** The homing process of HSPCs into the bone marrow niche. Created with BioRender.com.

A major advance in early BMOs research was the development of a 3D stromal-vascular co-culture system that allowed for an *in vitro* analysis of HSPC homing. Giger *et al.* presented a 3D matrix vascular co-culture system that allowed for an *in vitro* analysis of HSPC homing in their initial BMO studies (Figure 2) 17. HSPC homing is defined as the process by which transplanted HSPCs migrate to specific sites in the bone marrow and initiate engraftment and hematopoiesis 55. This process involves the stage wherein HSPCs migrate through the bloodstream; are implanted into the bone marrow microenvironmental; interact with various components, including MSCs and ECs; and settle in the bone marrow microenvironment 34, 56. ECs regulate the intravascular migration and initial adhesion of HSPCs on certain molecules, such as E-selectin and VCAM1 49, 57. In particular, when CXCL12 binds to CXCR4 on HSCs, integrin-mediated adhesion mechanisms, such as the activation of VLA-4-VCAM1 allowed HSPCs to stably attach to the vascular endothelium and migrate to the endothelium (Figure 2) 29, 56. This process is supported by the chemotactic and adhesive signals from MSCs and ECs 56, 58. In this context, early studies on the recreation of the bone marrow microenvironment have focused not only on interactions among MSCs, ECs, and HSPCs but also on the reconstruction of the microenvironment that is conducive to these interactions. The migration of HSPCs is dependent on the ratio of ECs and the degree of vascular-like network formation, which implies that the endothelial structure is a functional element regulating the migration of HSPCs beyond simple morphological features. A spatial analysis revealed that HSPCs migrate into the organoid and localize adjacent to the CD31^+^ and CD144^+^ endothelial networks. This is similar to the pattern of localization along *in vivo* vascular niches 59. Thus, the initial stromal-vascular co-culture model not only partially reproduces the bone marrow-like tissue structure but also simultaneously recapitulates the core functional features of HSPC homing.

### Transition to hiPSC-derived organoids

**Figure 3:**
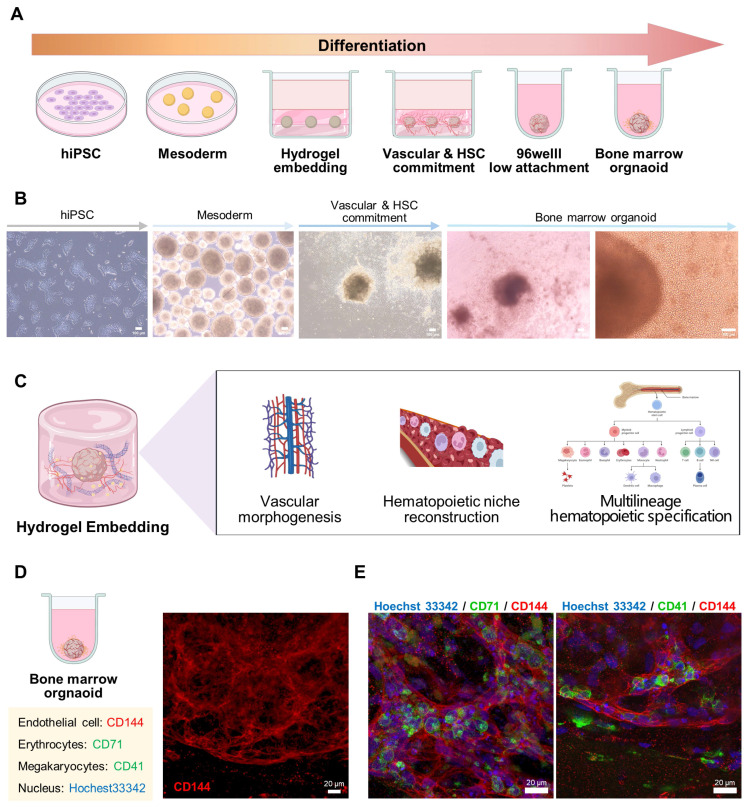
** Extracellular matrix-supported 3D culture system for vascular morphogenesis and multilineage hematopoietic specification. (A, B)** The differentiation of hiPSCs into BMOs. Scale bar = 100 μm.** (C)** These ECM combinations provide a microenvironment conducive to vascular morphogenesis and multilineage hematopoietic specification. **(D, E)** The immunofluorescence analysis further confirmed the co-organization of CD144^+^ ECs, CD71^+^ erythrocytes, and CD41^+^ megakaryocytes within the organoid. Scale bar = 20 μm. Created with BioRender.com.

Previous MSC-EC co-culture models marked an important step toward modeling the human hematopoietic microenvironment, but encountered several limitations in terms of long-term stability, scalability, and donor diversity 17. To address the limitations of early co-culture systems, recent studies have established standardized protocols for generating hiPSC-derived BMOs, providing reproducible and developmentally coherent platforms for hematopoietic niche reconstruction. This approach involves embedding hiPSC-derived embryoids by binding ECM components, such as collagen type 1 or 4, to Matrigel or Geltrex-like substrates and culturing them under 3D conditions (Figure 3) 4, 15. Consistent with this framework, our differentiation system also showed sequential morphological progression from early cellular aggregates to mature spheroid-like organoids during stepwise culture (Figure 3). Such composite matrix environments were determined as critical determinants for supporting vascular morphogenesis and multilineage hematopoietic specification (Figure 3). Moreover, the results of the immunofluorescence analysis confirmed that CD71^+^ erythrocytes and CD41^+^ megakaryocytes are organized around the organoid CD144^+^ ECs (Figure 3), which supports the concept that hiPSC-derived BMOs can spatially reproduce the cellular components of the hematopoietic microenvironment.

### Extracellular matrix guided self-organization and lineage balancing in bone marrow organoids

The ECM composition of the hiPSC-derived BMOs system plays a crucial role in regulating angiogenesis and hematopoietic lineage differentiation. Collagen type I provides a fibrous structural framework that supports tissue integrity and mechanical stability 60, 61. Collagen type IV, which is a major component of basement membranes, has been shown to create a microenvironment that is more permissive for bone marrow myeloid lineage differentiation 62, 63. Through comparative hydrogel studies, Khan *et al.* showed that a composite matrix combining collagen types I and IV with Matrigel improves angiogenesis compared with single-collagen conditions 4. Importantly, this combined ECM environment allows for the harmonious emergence of various myeloid–associated cell populations, including HSPCs, ECs, MSCs, megakaryocytes, erythrocytes, and monocytes 64. Similarly, Frenz-Wiessner *et al.* applied a strategy in which vascular organoids were embedded within hydrogels composed of collagen type I and Matrigel 15. Under these conditions, the persistence of ECs and the emergence of MSCs were confirmed. Single-cell analyses revealed distinct clusters corresponding to populations of ECs, hematopoietic cells, and MSCs, and confirmed that the hematopoietic population was further subdivided into myeloid populations and HSPCs. Collectively, these studies demonstrate that multiple myeloid-related cell populations can be harmoniously generated by incorporating mesenchymal-derived 3D cell aggregates into a specific hydrogel environment. These results support the concept that extracellular matrix composition plays not merely a structural role but also a directive function, inducing multicellular self-organization and promoting the reorganization of a functional perivascular hematopoietic microenvironment *in vitro*.

## Vascular architecture and niche specification in hiPSC-derived BMOs

### Structural reconstruction of the bone marrow vascular network

**Figure 4:**
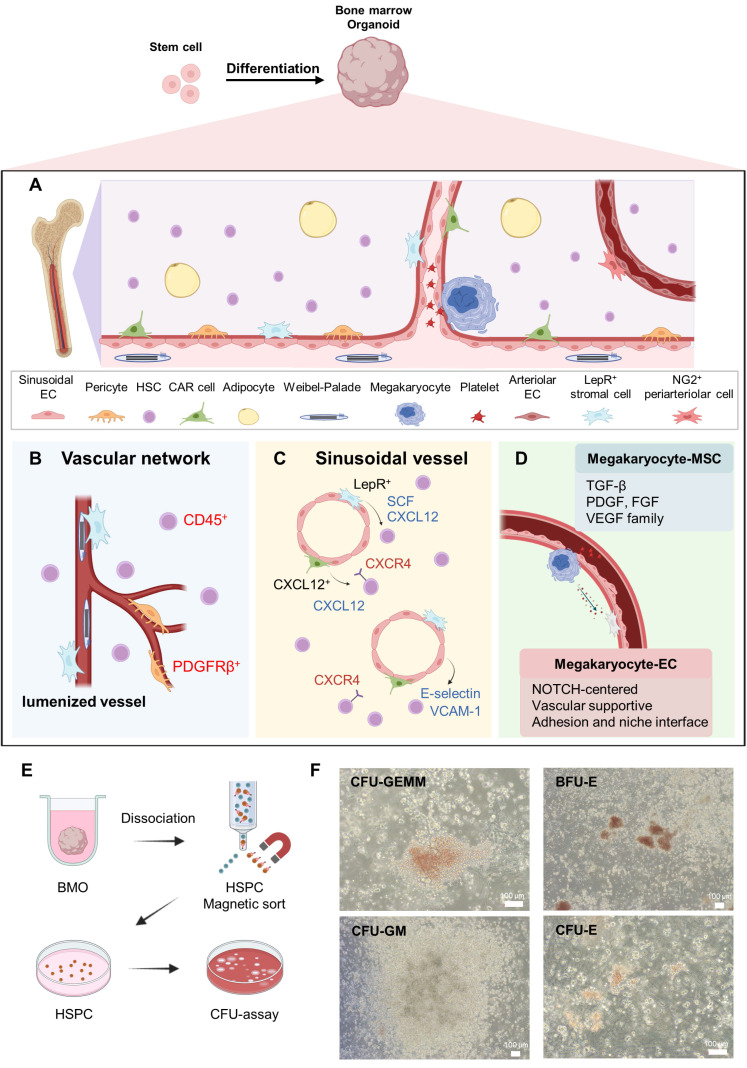
** Structural and functional features of the bone marrow organoid vascular niche. (A, B)** The bone marrow vascular microenvironment and its major cellular components, including lumenized vessels, PDGFRβ^+^ perivascular cells, and surrounding CD45^+^ HSPCs. **(C)** The sinusoidal vessels form a key hematopoietic niche in which LepR^+^ and CXCL12^+^ stromal populations provide SCF and CXCL12, while E-selectin, VCAM-1, and CXCR4-mediated signaling regulate the localization and retention of HSPCs. (**D**) Thrombopoiesis-related signaling in the perisinusoidal niche. **(E, F)** A CFU assay was used to assess the hematopoietic potential of HSPCs isolated from BMOS, along with the representative images of the CFU-GEMM, BFU-E, CFU-GM, and CFU-E colonies. Scale bar = 100 μm. Created with BioRender.com.

Recent studies have established that HSPCs reside in the perivascular regions of the native bone marrow, wherein endothelial and stromal components cooperate to regulate stem cell maintenance and differentiation (Figure 4). In hiPSC-derived BMOs, a network of lumenized vascular structures was established throughout the organoid, with hematopoietic cells distributed both within and surrounding the endothelial-lined vessels 4, 15. The perivascular distribution of CD45⁺ cells in organoids suggests the *in vitro* functional reconstruction of a hematopoietic microenvironment. Evidence of vascular maturation was further supported by platelet-derived growth factor receptor-β (PDGFRβ) positive perivascular cells enveloping endothelial structures, along with the formation of lumenized vessels. PDGFRβ expression is characteristic of pericytes and stromal subsets that contribute to vascular stabilization and endothelial survival (Figure 4) 65, 66. The presence of Weibel–Palade bodies within ECs further indicates acquisition of functional endothelial identity (Figure 4) 67. Collectively, these findings suggest that BMOs reconstruct a structurally organized vascular network resembling the architecture of the native bone marrow.

#### Perivascular stromal organization and formation of CXCL12 abundant reticular cell like niches

The perivascular niche represents a specialized microenvironment surrounding the bone marrow vasculature that regulates the maintenance, quiescence, and trafficking of HSCs 68, 69. This niche constitutes stromal populations, including LepR^+^ MSCs, which support hematopoiesis via the secretion of key factors such as CXCL12 and SCF (Figure 4) 69. In hiPSC-derived BMOs, Frenz-Wiessner *et al.* reported that LepR^+^ cell populations were localized adjacent to vascular structures, indicating the incorporation of a stromal compartment within the reconstructed vascular niche 15. In the native bone marrow, HSPCs primarily reside in the perivascular region, where coordinated interactions between endothelial and stromal cells regulate their maintenance and differentiation 25, 70, 71. Thus, the perivascular spatial organization of the MSC subsets in BMOs recapitulates the key structural features of the physiological HSC niche. These stromal cells represent the key components of the HSC niche and support hematopoietic maintenance through CXCL12-mediated signaling 29, 33. Their spatial organization around vascular networks indicate the integration of an MSCs compartment within the vascular niche 2, 25. Moreover, CXCL12^+^ cells tend to form a reticular network structure along the vascular structures, supporting the formation of CAR cell-like populations. In the naïve bone marrow, CAR cells are distributed around the HSCs and regulate their establishment, quiescence, and spatial localization through the CXCL12-CXCR4 signaling axis 29, 31, 32. Within the perivascular niche, CXCL12-CXCR4 signaling plays a key role in maintaining HSC dormancy and inducing position maintenance 29. Thus, the presence of a similar CXCL12^+^ reticular network in BMOs implies a functional reconfiguration of the CAR-related perivascular microenvironment.

#### Endothelial patterning and sinusoidal features driven by vascular endothelial growth factor C (VEGFC)

The sinusoidal ECs in the bone marrow are characterized by high permeability and a relatively elevated ROS environment and secrete several factors such as CXCL12, VCAM1, and E-selectin to promote the activation, regeneration, and differentiation of HSCs 51, 52. These vascular endothelial subtypes establish distinct microenvironmental niches that differentially regulate the localization and functional states of HSCs. Khan *et al.* suggested that the addition of VEGFC during organoid differentiation reconstituted a sinusoidal-like bone marrow microenvironment *in vitro*, recapitulating key molecular and functional features of sinusoidal ECs 4. The transcriptional profiling of these conditions revealed an overall upregulation of VEGF receptors, including FLT4, which encodes VEGFR3, a marker that is abundant in sinusoidal ECs 72, 73.

Simultaneously, the expression of HSC adhesion molecules, including VCAM1 and integrin subunit alpha 4 (ITGA4), was increased, as was the expression of HSC support growth factors and chemotactic cytokines such as CXCR4 and FGF4 4. Collectively, the sinusoidal-like vascular niche is enhanced in BMOs, and the structural and functional characteristics that support the adhesion, retention, migration, and survival of HSPCs are evident 29, 74. These organoids form lumenized vascular networks containing hematopoietic cells, and HSPCs are mainly localized in the perivascular regions. Megakaryocytes were observed adhering to endothelial barriers and extending proplatelets into the vascular lumen, which accurately reflects an important aspect of platelet production in the bone marrow 75. Taken together, these findings suggest that BMOs support vascular morphogenesis and reconstruct a structurally organized and functionally competent sinusoidal-like microenvironment capable of sustaining hematopoietic regulation.

#### Crosstalk among megakaryocytes endothelial cells and stromal cells within the perivascular niche

Khan *et al.* reported that hiPSC-derived BMOs recapitulate the major structural and molecular interactions among megakaryocytes, ECs, and MSCs within the bone marrow microenvironment 4. In particular, megakaryocytes were observed in proximity to the endothelial barrier, suggesting that the organoid system recapitulates the spatial architecture of the perivascular microenvironment. This spatial arrangement is also consistent with the physiological context in the bone marrow, where megakaryocytes closely associate with arterial endothelium and extend proplatelets toward the vascular lumen during platelet production (Figure 4) 76, 77. Consistent with these structural features, extensive receptor-ligand interactions were identified among megakaryocytes, ECs, and MSCs, indicating that the BMOs model not only maintains hematopoietic cell maintenance but also dynamic intercellular communication within the stromal microenvironment. The bidirectional signaling axes detected between megakaryocytes and ECs could be functionally categorized into Notch-centered cell state regulatory signaling, vascular supportive signaling involving the VEGF/ ANGPT/ PDGF/ KITLG pathways, and signaling associated with adhesion and niche interface regulation, including SELP-CD34 and FGF2-CD44 4, 54, 78-80. These signals suggest that direct contact with ECs regulates megakaryocyte positioning, maintenance, survival, and functional maturation within the vascular niche 81, 82. In addition, the increased expression of FLT4, ANGPT2, and DLL4 demonstrate that the arterial endothelial program is enhanced in organoid ECs. These vascular characteristics serve as an important basis for the localization of perivascular megakaryocytes and the formation of a microenvironment suitable for platelet production 4, 83. Collectively, these interactions suggest that ECs may provide instructive cues that regulate the positioning, maintenance, survival, and functional maturation of megakaryocytes within the vascular microenvironment. Meanwhile, the interactions between MSCs and megakaryocytes were centered on TGF-β, PDGF, FGF, and VEGF family members and their corresponding receptors 4. The growth factors such as PDGF, TGF-β, and FGF stored in the α-granules of megakaryocytes can regulate the state and differentiation potential of MSCs, while MSCs can respond to these signals to provide a substrate environment conducive to the maintenance and maturation of megakaryocytes 76, 84. Collectively, these findings suggest that BMOs recapitulate essential features of the megakaryocyte-associated vascular microenvironment and suggest that this platform may also support microenvironmental programs relevant to thrombopoiesis.

### Myeloid lineage differentiation

The human bone marrow contains HSPCs that can differentiate into multiple blood cell lineages 64. Intramedullary HSPCs can potentially mature along the myeloid differentiation pathway via common myeloid progenitor cells 23, 64. This is precisely tuned by interactions in the bone marrow niche, and for BMOs to reflect the physiological functions of the human bone marrow, it must be possible to reproduce not only the presence of HSPCs but also the ability to differentiate into various myeloid lineage progenitors. Khan *et al.* suggested that aside from merely containing HSPCs, BMOs can also structurally and functionally recapitulate the key aspects of myeloid differentiation observed in the human bone marrow 4. Specifically, bone marrow myelomonocytic, megakaryocytic, and erythroid cells were identified within the organoids, and the differentiation trajectory analysis showed that major differentiation axes, including the megakaryocyte–erythroid and monocyte–neutrophil lineages, were reproduced 4, 23, 85. This suggests that BMOs can serve as a significant model reflecting the myeloid differentiation program of the human bone marrow. Moreover, Park *et al.* isolated BMO-derived HSPCs and assessed their functional potential using a colony-forming unit (CFU) assay 18. The results demonstrated the differentiation into erythroid, granulocytic, and monocyte/macrophage lineages, validating the usefulness of BMOs as a platform for assessing both myeloid differentiation capacity and HSPC function 86. In concordance with these reports, the CFU assays performed on the cells dissociated from our BMOs yielded multiple colony subtypes, including colony-forming unit-granulocyte, erythrocyte, monocyte, and megakaryocyte (CFU-GEMM), burst-forming unit-erythroid (BFU-E), colony-forming unit-granulocyte/macrophage (CFU-GM), and colony-forming unit-erythroid (CFU-E) (Figure 4), further confirming the multilineage progenitor potential of organoid-derived hematopoietic cells.

### Lymphoid lineage differentiation

Stem cells derived from the bone marrow are important immune organs that produce and mature white blood cells and various immune cells that are at the core of the immune response 87. Within the bone marrow, immune cells, including T cells, B cells, neutrophils, dendritic cells, and NK cells, closely interact with HSCs to maintain HSC function and regulate inflammatory responses and tissue repair 4, 15, 18, 87. A recently developed BMOs system has successfully reconstructed these complex immune cell lineages and niche interactions in a physiologically relevant architecture 15, 18. In the BMOs, both myeloid and lymphoid progenitor cells differentiated, and major immune cells such as monocytes, macrophages, neutrophils, basophils, eosinophils, and mast cells, were observed in a compartmentalized form 15, 18. This suggests that BMO is a model that reflects the cellular composition of multilineage hematopoiesis. Frenz-Wiessner *et al.* reported that BMOs faithfully recapitulate granulopoiesis, indicating a sequential maturation of neutrophil precursors into immature and mature neutrophils closely resembling those in the human bone marrow 15. Notably, this process occurred without the exogenous G-CSF, suggesting that intrinsic niche cues regulate myeloid maturation. Upon LPS stimulation, BMOs exhibited increased secretion of inflammatory cytokines IL-6, IL-8, and G-CSF, accompanied by expansion of the preNeu population, effectively modeling human-like emergency granulopoiesis 15. Furthermore, the potential for T-cell lineage differentiation was confirmed in an artificial thymic organoid (ATO) environment constructed using BMO-derived HSCs and MS5-DLL4, demonstrating that the organoid-based microenvironment enables lymphoid and myeloid differentiation. These research results suggest that BMOs can be a potential research platform for diseases induced by immune cells. This functional responsiveness is useful for studying the pathophysiology of diseases caused by unregulated immune-stromal interactions. Furthermore, in bone marrow diseases characterized by the abnormal signaling between immune and stromal cells, organoids can incorporate patient-derived cells to recapitulate inflammatory cytokine production, stromal reorganization, and immune-stromal dysfunction 88.

## *In vivo* evaluation of bone marrow organoids

### Structural engraftment of intact bone marrow organoids

Ren *et al.* transplanted intact BMOs beneath the kidney capsule of immunodeficient NSG mice to determine whether these organoids can sustain long-term structural and functional stability within a physiological environment 16. The subcapsular kidney space has a rich vascular distribution and excellent graft survival 89. A histological examination conducted 1 month after transplantation confirmed that the organoids had successfully integrated into the host tissue, with clear evidence of engraftment beneath the renal capsule and the presence of vascular lumens surrounding the erythrocytes. The formation of vascular lumens in the mouse model suggests the capacity for physiological hematopoietic maintenance and confirms the potential for intercellular niche communication. Flow cytometric monitoring further revealed the persistent presence of human CD45^+^ leukocytes and CD235a^+^ erythroid cells for up to 11 months, indicating the long-term survival and hematopoietic activity of the transplanted BMOs *in vivo*. These findings underscore BMOs. These findings underscore the significance of BMOs as a platform capable of achieving long-term vascular integration and sustaining multilineage hematopoiesis following transplantation.

### Hematopoietic reconstitution by bone marrow organoids-derived HSPCs

Frenz-Wiessner *et al.* isolated CD34^+^ cells from BMOs by FACS and intravenously injected them into NSG-HLA-DQ8 mice. Serial peripheral blood analyses detected human CD45^+^ cells at weeks 5–10 post-transplantation, indicating partial engraftment 15. Analysis of the bone marrow niche demonstrated that the transplanted cells survived for 10–12 weeks, with some animals exhibiting increased proportions of human CD45^+^ cells over time. Furthermore, the engrafted human cells differentiated into multiple hematopoietic lineages, including CD20^+^ B cells and CD33^+^ myeloid cells. These findings suggest that BMO-derived HSPCs possess functional hematopoietic reconstitution capacity *in vivo*.

### Therapeutic potential of bone marrow organoid based transplantation

Park *et al.* generated BMOs from hiPSCs and reported that this model can replicate the key elements of the human bone marrow microenvironment 18. The generated BMOs formed an organized stromal-vascular structure and proved the maintenance and differentiation of hematopoietic progenitor cell populations. These suggest that the BMOs successfully replicated the key elements of the functional human bone marrow microenvironment. In subsequent studies, the research team assessed the potential of BMOs for regenerative therapy through *in vivo* transplantation experiments using immunodeficient mice. After establishing a model of severe bone marrow injury by irradiating NSG mice with gamma rays, BMOs were transplanted into the irradiated recipients, and the recovery of the hematopoietic system was analyzed. As a result, the survival rates improved in the transplant group, and the partial engraftment of human hematopoietic cells was confirmed. Collectively, this study confirmed the potential of BMO-based bone marrow structures as a therapeutic platform that not only reproduces the pathological characteristics of the human hematopoietic microenvironment but also supports the regeneration of the damaged hematopoietic system.

## Disease modeling of bone marrow organoids

### Myelofibrosis: pathological basis

**Figure 5:**
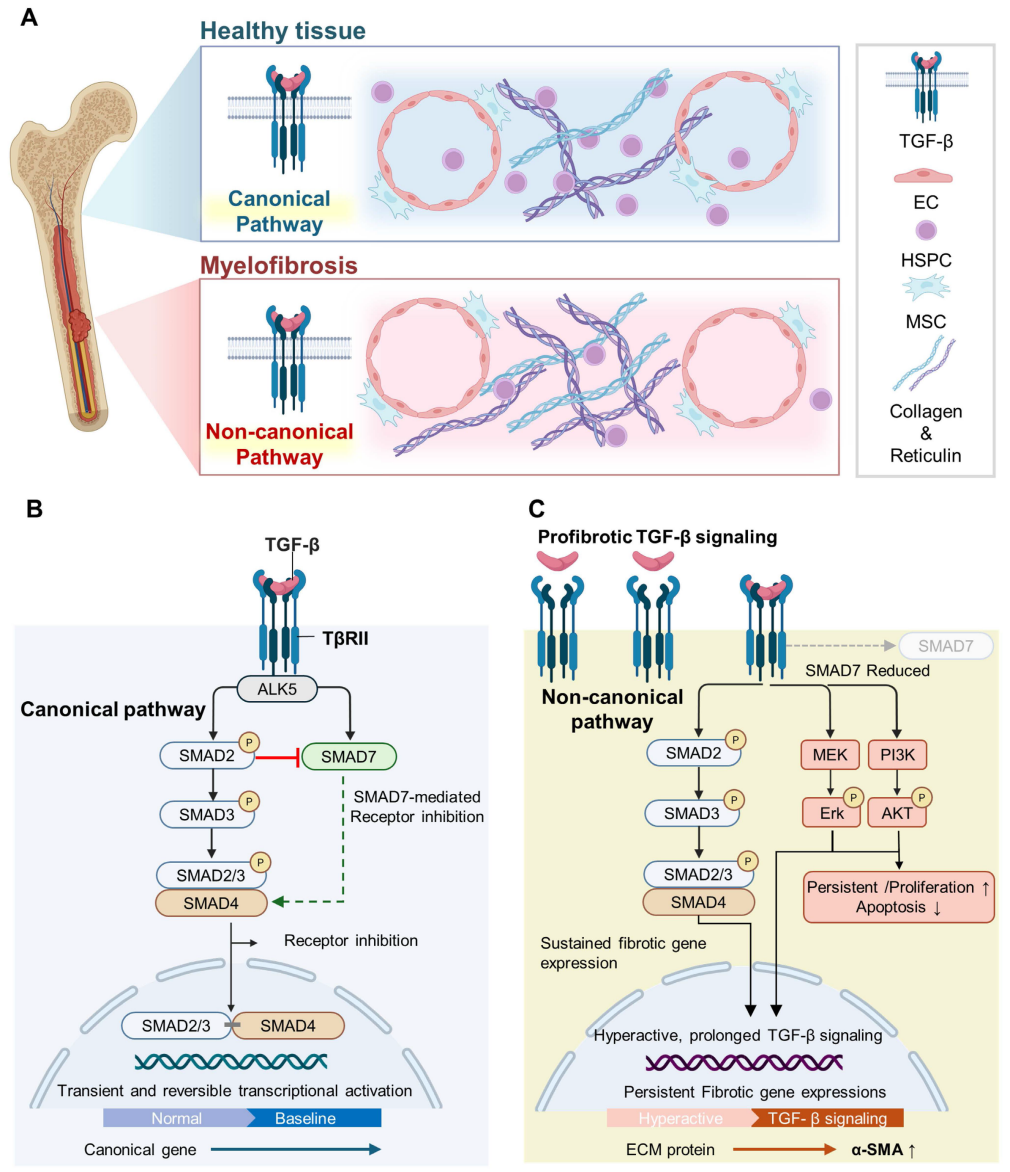
** Canonical and non-canonical TGF-β signaling in healthy bone marrow and myelofibrosis. (A)** The differential regulation of TGF-β signaling in a normal bone marrow and myelofibrosis. Myelofibrosis is characterized by the accumulation of fibrotic fibers, including collagen and reticulin, together with a reduction in HSPCs within the bone marrow niche. **(B)** In the normal bone marrow, TGF-β signaling is tightly regulated, leading to transient and reversible transcriptional activation that maintains tissue homeostasis. **(C)** In myelofibrosis, non-canonical TGF-β signaling becomes dominant, accompanied by reduced SMAD7-mediated inhibition, prolonged pathway activation, and persistent fibrotic gene expression. Created with BioRender.com.

Myelofibrosis, a hematological malignancy, is characterized by an excessive accumulation of reticulin and collagen in the bone marrow microenvironment (Figure 5) 90, 91. A central driver of marrow fibrosis is the sustained activation of TGF-β signaling 91. Activated TβRII subsequently recruits the type I receptor ALK5 to form a functional receptor complex 91, 92. Upon complex formation, ALK5 phosphorylates and activates SMAD2/3, allowing for these regulatory SMADs to associate with SMAD4 and translocate into the nucleus, where they modulate the transcription of fibrosis-related target genes (Figure 5) 92, 93. The activation of this canonical, SMAD-dependent TGF-β signaling pathway drives the differentiation of fibroblasts into α-SMA–expressing myofibroblasts and markedly increases the production of ECM components, including collagen type I/III and fibronectin 91, 94. The inhibitory SMAD7 attenuates receptor activity and limits SMAD2/3 nuclear signaling, maintaining ECM homeostasis (Figure 5) 92, 95. In myelofibrosis, sustained TGF-β signaling and impaired negative feedback result in prolonged SMAD activation, persistent fibrotic transcriptional programs, and excessive ECM deposition 92, 96. The resulting excessive matrix deposition and increased microenvironmental stiffness amplify the inflammatory signaling and progressively disrupt the hematopoietic niche, ultimately impairing normal hematopoiesis and contributing to the development and progression of bone marrow fibrosis 97.

### Modeling acquired myelofibrosis using bone marrow organoids

Khan *et al.* treated BMOs with TGF-β to model the pathological process of myelofibrosis *in vitro* 4. This stimulation significantly increased the fibrosis-related markers such as α-SMA, IL-11, and COL1A1, and induced the major molecular changes of myelofibrotic progression within BMOs 4, 98, 99. Importantly, fibrosis marker expression was significantly reduced when TGF-β signaling was inhibited with SB431542 or when treated with the bromodomain inhibitor JQ1 100, 101. This suggests that the model is not merely a simulation of pathology but can be used as an experimental platform to assess responses to antifibrotic therapy. Beyond induced fibrosis, the researchers further assessed the engraftment capacity of patient-derived HSPCs within BMOs 4. Both healthy donor and myelofibrosis patient-derived HSPCs successfully engrafted and interacted with the stromal matrix. The organoids seeded with patient-derived cells showed an increased deposition of fibrotic factors, confirming disease-relevant remodeling at the tissue level. Furthermore, they reported that mutant hematopoietic clones can preserve clonal heterogeneity within organoids and mimic malignant stem cell niches *in vitro*, highlighting the potential of BMOs for disease-specific microenvironmental interactions and therapeutic studies in hematological malignancies.

### Modeling VPS45 associated inherited myelofibrosis using bone marrow organoids

BMOs were used to model hereditary myelofibrosis associated with *VPS45* deficiency 15. *VPS45* mutations are clinically associated with severe congenital neutropenia, progressive bone marrow failure, and myelofibrosis 102, 103. Frenz-Wiessner *et al.* established BMOs from iPSCs carrying a homozygous *VPS45 Thr224Asn* mutation and observed stromal abnormalities resembling myelofibrosis, including proliferation of α-SMA–positive myofibroblast-like cells and increased reticulin deposition confirmed via Gomori staining 15. Moreover, altered granulopoiesis, with increased mature neutrophil populations, was detected in mutant organoids. *VPS45* mutant BMOs are characterized by reticular fibrillar deposition and an expanded population of α-SMA-expressing stromal cells, consistent with the patient biopsy findings 15, 99, 102. Collectively, these studies suggest that hiPSC-derived BMOs can model both acquired and inherited myelofibrosis, can be used in mechanistic studies of fibrotic transformation, and can provide a platform for therapeutic evaluation.

### Modeling radiation induced hematopoietic syndrome using bone marrow organoids

**Figure 6:**
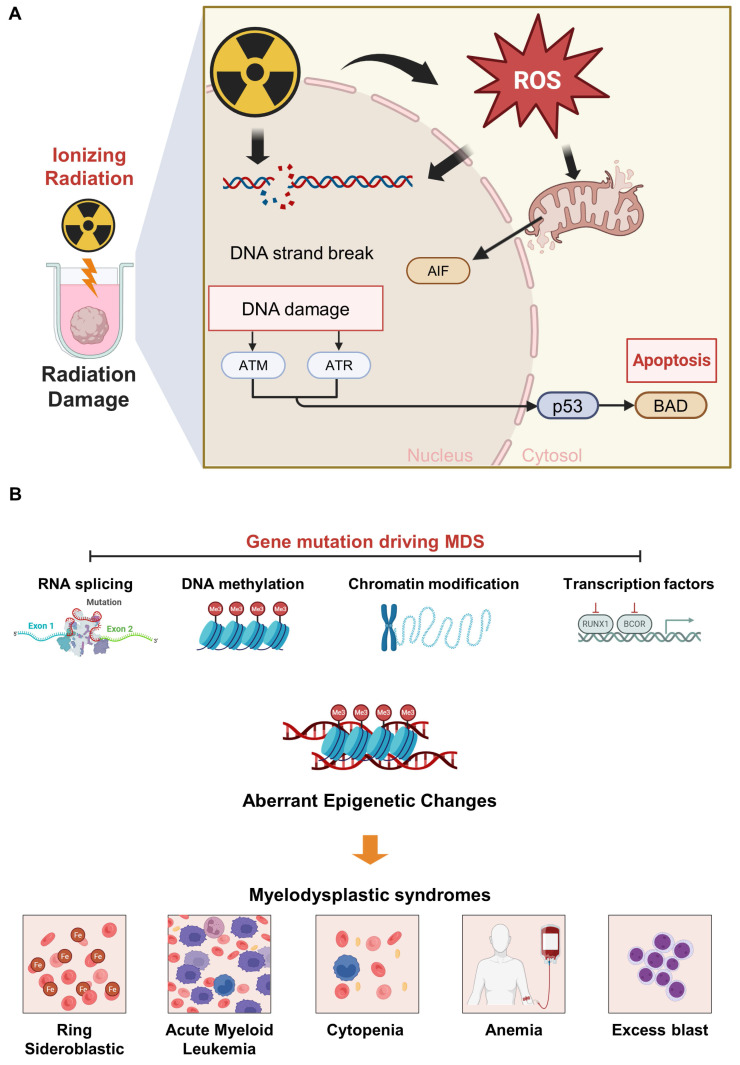
Mechanisms underlying radiation-induced hematopoietic syndrome and gene mutation-driven myelodysplastic syndrome (MDS). (A) ROS generation and DNA damage induced by ionizing radiation activate the ATM, ATR, and p53 pathways, leading to mitochondrial dysfunction and apoptosis, thereby mediating radiation-induced hematopoietic injury. (B) Gene mutations involving aberrant splicing, DNA methylation, chromatin modification, and transcription factor dysregulation disrupt hematopoiesis and ultimately give rise to the major clinical features of MDS. Created with BioRender.com.

Hematopoietic syndrome is a radiation-induced disorder resulting from the depletion of HSPCs within the bone marrow following exposure to high-dose ionizing radiation 104, 105. Ionizing radiation not only directly induces DNA double-strand breaks but also generates excessive ROS, thereby resulting in additional base lesions, single-strand breaks, and chromosomal instability (Figure 6) 104, 106. The accumulation of DNA damage induces replication stress in proliferating HSPCs, promoting replication fork stalling and collapse, thereby resulting in the excessive activation of the ATM/ATR– p53–mediated DNA damage response 107, 108. This damage results in the severe impairment of marrow function, which manifests as rapid declines in leukocytes, lymphocytes, and other blood cell populations, increasing susceptibility to infection, hemorrhage, and immune dysfunction 108, 109. Park *et al.* exposed BMOs to ionizing radiation at doses of 3, 6, and 9 Gy to model this condition *ex vivo* 18. The irradiated organoids showed structural disintegration, closely resembling the architectural collapse observed in irradiated bone marrow *in vivo*. Correspondingly, accompanied by the increased expression of apoptotic and DNA damage markers, including phosphorylated γH2AX, BAD, and AIF, as well as elevated numbers of *TUNEL*-positive cells 18. Notably, the expression of AIF and BAD, which promotes mitochondrial membrane permeabilization was significantly upregulated, confirming radiation-induced cytotoxic and genotoxic 110, 111. These findings establish the BMO as a physiologically relevant platform for modeling radiation-induced hematopoietic failure, offering potential applications in studying marrow regeneration and radioprotective therapeutic strategies.

### Myelodysplastic syndrome

Myelodysplastic syndromes (MDS) are a group of clonal hematopoietic disorders driven by somatic mutations in HSCs, resulting in ineffective hematopoiesis, increased apoptosis, and peripheral cytopenias 112. These defects cause cytopenias, including anemia, neutropenia, and thrombocytopenia 113, 114. In MDS, recurrent mutations commonly involve genes that can be broadly classified into several functional categories, including RNA splicing, DNA methylation, chromatin modification, and transcription factors, all of which contribute to disordered hematopoietic differentiation and clonal expansion (Figure 6 and Table 2) 115, 116. The gene mutations commonly associated with MDS include *TET2*, *DNMT3A*, *TP53*, *SF3B1*, *ASXL1*, *U2AF1*, and several additional genes, among others 117. Ren *et al.* modeled MDS using BMOs seeded with CD34^+^ HSCs obtained from three patients harboring distinct genetic mutations and clinical phenotypes 16. *TET2* mutations, clinically present with mild anemia without marked leukopenia or thrombocytopenia, a pattern that is characteristic of some *TET2*-mutated cases. *TET2* mutations result in hematopoietic dysfunction by decreasing DNA demethylation activity and resulting in the persistence of a stem cell-like state with impaired differentiation 112, 115, 118. *SF3B1* mutation induces spliceosome dysfunction resulting in abnormal mitochondrial iron accumulation, ring sideroblast formation, and defective erythropoiesis—a hallmark phenotype associated with this mutation 118-120. These defects interfere with normal red blood cell maturation and act as a key cause of persistent anemia in *SF3B1*-mutant MDS. *U2AF1* mutations also cause extensive abnormalities throughout RNA splicing, which consequently disrupts inflammatory pathways, membrane protein expression, and hematopoiesis-related signaling 118, 121. As a result, lineage differentiation is impaired, resulting in the accumulation of immature progenitors and clinical manifestations such as anemia, multilineage dysplasia, and leukocytosis 122. The patient-derived CD34⁺ cells were successfully engrafted within BMOs and maintained over time. These organoids reproduced cytopenia and hematopoietic dysfunction as observed in the MDS patients, suggesting that BMOs can reflect patient-specific hematopoietic defects. The exome DNA sequencing results showed that the patient-specific mutant clones within the organoids maintained similar allele frequencies *in vivo*. This implies that BMOs can also be used to study somatic mutation maintenance and clonal evolution. Consequently, these MDS organoid models can potentially serve as precision disease modeling platforms for patient-specific genetic and functional pathophysiological studies, the analysis of hematopoietic microenvironmental changes, and validation of novel therapeutic targets.

## Conclusions and future perspectives

hiPSC-derived BMO models can be used as physiologically relevant *in vitro* models that recapitulate the human bone marrow microenvironment to study hematopoietic development and hematopoietic diseases. This approach reduces the reliance on immunodeficient mouse models and provides a platform for the efficient transplantation and maintenance of HSCs derived from patients with diverse disease backgrounds, allowing for the investigation of patient-specific disease characteristics 123, 124. By generating and isolating HSPCs within BMOs and validating their multilineage differentiation potential, functional studies can be conducted using HSPCs generated within the organoid system itself 18. This capability represents a significant advance in the study of the mechanisms of human hematopoiesis and the pathophysiology of blood diseases. Furthermore, by faithfully recapitulating the bone marrow microenvironment, the use of BMOs allows for the in-depth analysis of stem cell niche regulation and associated molecular pathways. Specifically, these systems facilitate the study of cell-to-cell and cell-to-extracellular matrix interactions that regulate HSC behaviors within the bone marrow microenvironment.

One future application of BMO is the development of personalized BMOs. Since BMOs can be generated from patient-derived stem or progenitor cells, they provide the advantage of reproducing individual disease phenotypes within physiologically relevant microenvironments 125. When patient-derived HSPCs with different genetic mutations are transplanted into BMOs, it is possible that disease-specific characteristics associated with each mutation will be reproduced within the organoid 16. Furthermore, it has been reported that when HSPCs derived from myelofibrosis patients are transplanted into BMOs, disease characteristics related to fibrosis are reproduced within the organoid microenvironment 4. Thus, such patient-specific platforms can provide significant advantages to precision medicine by allowing for the prediction of individual drug responses, the identification of disease-causing mechanisms, and the identification of new therapeutic targets.

Another major application of BMOs is drug screening. Because these systems better mimic the human bone marrow microenvironment than conventional two-dimensional culture models, they provide a more predictable platform for evaluating therapeutic efficacy and toxicity 126. Furthermore, BMOs can reduce the time and cost of preclinical evaluation by enabling rapid screening of large drug libraries in a patient-specific manner. Indeed, fibrosis BMOs models have already demonstrated responsiveness to therapeutic interventions, with TGF-β inhibitor treatment reducing fibrosis-related phenotypes 4. These results demonstrate the potential of BMOs as a translational platform for testing therapeutic agents in hematological and bone marrow diseases.

Artificial intelligence (AI)-assisted prediction is also likely to become an important future direction in BMOs research. By integrating imaging, transcriptomic, and functional readouts from BMOs, AI-based approaches may improve the prediction of drug responses and enhance precision medicine strategies 127-129. Moreover, these models may help identify early events involved in the transition from normal hematopoiesis to pathological hematopoiesis and predict whether specific mutational combinations are likely to drive disease progression within the bone marrow microenvironment. AI could also be applied to organoid quality control, including the prediction of organoid maturation, HSPCs maintenance capacity, and experimental success rates based on early morphological or functional features, ultimately helping to reduce batch-to-batch variability 129.

Finally, future studies may extend beyond BMOs alone toward integrated BMO systems. One possible direction is the development of models incorporating an osteogenic outer scaffold or shell that mimics the structural properties of the bone, with an inner marrow-like compartment containing hematopoietic and stromal elements 130. Another approach would be to co-culture BMOs with osteogenic cell populations to better reproduce endosteal and marrow niche interactions. Such integrated bone–marrow models may better recapitulate the *in vivo* relationship between bone and marrow and further improve the physiological relevance of organoid-based systems. Currently, BMO-based disease modeling studies are limited to a few disease groups, such as myelofibrosis, myelodysplastic syndrome, and radiation-induced hematopoietic syndrome. BMO systems may also be expanded as platforms for studying the pathophysiology of a broader range of bone marrow diseases. For example, bone marrow failure syndromes, in which the bone marrow fails to produce sufficient blood cells, and infectious and inflammatory bone marrow diseases could become targets for future BMO-based modeling and analysis 131. These diseases involve the complex dysregulation of the bone marrow environment, including altered hematopoietic signaling, immune responses, and interactions between hematopoietic and stromal cells 132. Thus, BMO platforms could be further developed to model a broader range of bone marrow disorders beyond the disease settings currently studied. In conclusion, BMOs have rapidly evolved into versatile platforms for modeling hematopoiesis, bone marrow pathology, and patient-specific disease features. Furthermore, continued advances in personalized organoid design, drug screening, AI-assisted prediction, and integrated bone–marrow modeling are expected to further expand their value in basic research, translational studies, and regenerative medicine.

## Figures and Tables

**Table 1:** ** Bone marrow-derived hematopoietic cell lineages, markers and functional characteristics.** The representative bone marrow-derived hematopoietic cell populations, their commonly used surface markers, and major functional characteristics.

Lineage	Cell	Markers	Features	References
Myeloid lineage	Neutrophils	CD15^+^, CD11b^+^, CD66b^+^	Eliminate bacterial infection	133, 134
	Eosinophils	CCR3⁺, CD125^+^	Allergic reaction	135, 136
	Basophils	CD125^+^, CD193⁺	Allergic reaction	137
	Monocytes	CD14^+^, CD16^+^	Differentiation into macrophages	138
	Macrophages	CD68⁺, CD163^+^	Phagocytosis, antigen processing	139
	Megakaryocytes	CD41^+^, CD42b^+^	Platelet production	140
	Erythrocytes	CD235a^+^	Red blood cell maturation	141
	Platelets	CD41^+^, CD61^+^, CD42b^+^	Hemostasis	142, 143
	Dendritic cells	CD11c⁺, MHC-II⁺	Antigen presentation	144
Lymphoid lineage	B cells	CD19^+^, CD20^+^	Antibody production	145
	T cells	CD3⁺	Cellular immunity	146
	NK cells	CD56⁺, CD16⁺	Nonspecific killing	147
	Plasma cells	CD38^+^, CD138^+^	Antibody production	148

**Table 2:** ** Gene mutations in myelodysplastic syndromes.** The table summarizes recurrent mutations in MDS, categorized by their principal biological functions, including RNA splicing, DNA methylation, chromatin modification, and transcriptional regulation. These mutations contribute to ineffective hematopoiesis, cytopenia, multilineage dysplasia, ring sideroblast formation, and progression to AML.

Functional category	Gene	Primary function	Clinical significance in MDS	Reference
RNA splicing	*SF3B1*	Core spliceosome component required for branch point recognition and accurate pre-mRNA splicing.	Abnormal mitochondrial iron accumulation, ring sideroblasts, and defective erythropoiesis	116, 149-153
	*SRSF2*	Regulates sequence-specific exon recognition via binding to exonic splicing enhancer motifs.	High AML progression	
	*U2AF1*	Essential splicing factor that recognizes the 3′ splice site during pre-mRNA splicing to ensure functional mRNA generation.	Anemia, multilineage dysplasia, and leukocytosis	
DNA methylation	*TET2*	Regulates DNA demethylation to maintain proper HSC self-renewal and lineage differentiation.	Mild anemia without marked leukopenia or thrombocytopenia	116, 154, 155
	*DNMT3A*	Functions as a DNA methyltransferase responsible for *de novo* DNA methylation.	Higher risk of leukemia transformation	
	*IDH2*	Inhibits TET2-dependent demethylation by generating 2-hydroxyglutarate.	Mild anemia without marked leukopenia	
Chromatin modification	*ASXL1*	Controls chromatin remodeling via histone modification regulation.	Clonal hematopoiesis, myeloid malignancies	116, 156, 157
	*EZH2*	Encodes a core component of the PRC2 complex.	Anemia, thrombocytopenia, and neutropenia	
Transcription factors	*RUNX1*	Hematopoietic transcription factor required for normal blood cell development and differentiation.	Multilineage dysplasia, cytopenia with excess blasts and thrombocytopenia	116, 158-160
	*BCOR*	Epigenetic corepressors required for transcriptional repression and regulation of myeloid cell proliferation and differentiation	multilineage dysplasia and cytopenia with excess blasts	

## Abbreviations

CFU-F: colony-forming unit-fibroblast
LTBMC: long-term bone marrow culture
LTHR: long-term hematopoietic reconstitution
CAR: CXCL12-abundant reticular
SCF: stem cell factor
3D: three-dimensional
NK: natural killer
ECM: extracellular matrix
ANG-1: angiopoietin-1
Tie2: TEK receptor tyrosine kinase
VCAM1: vascular cell adhesion molecule 1
LepR: leptin receptor
Nestin: neuroepithelial stem cell protein
VLA-4: very late antigen-4
PDGFRβ: platelet-derived growth factor receptor β
VEGFR3: vascular endothelial growth factor receptor 3
ITGA4: integrin subunit alpha 4
FGF4: fibroblast growth factor 4
ANGPT: angiopoietin
PDGF: platelet-derived growth factor
KITLG: KIT ligand
FGF2: fibroblast growth factor 2
ANGPT2: angiopoietin 2
TGF-β: transforming growth factor-β
CFU: colony-forming unit
ATO: artificial thymic organoid
TβRII: transforming growth factor-β type II receptor
ALK5: activin receptor-like kinase 5
α-SMA: alpha-smooth muscle actin
IL-11: interleukin-11
VPS45: vacuolar protein sorting 45 homolog
p53: protein 53
γH2AX: phosphorylated gamma H2AX
BAD: BCL2-associated agonist of cell death
AIF: apoptosis-inducing factor
TUNEL: terminal deoxynucleotidyl transferase dUTP nick-end labeling
MDS: myelodysplastic syndromes
DNMT3A: DNA methyltransferase 3 alpha
TP53: tumor protein p53
